# Untargeted metabolomic analysis of thoracic blood from badgers indicate changes linked to infection with bovine tuberculosis (*Mycobacterium bovis*): a pilot study

**DOI:** 10.1007/s11306-022-01915-6

**Published:** 2022-07-27

**Authors:** James Scott Bauman, Richard Pizzey, Manfred Beckmann, Bernardo Villarreal-Ramos, Jonathan King, Beverley Hopkins, David Rooke, Glyn Hewinson, Luis A. J. Mur

**Affiliations:** 1 Institute of Biological, Environmental and Rural Science, Aberystwyth University, Aberystwyth, SY23 3DA Ceredigion UK; 2 Centre of Excellence for Bovine Tuberculosis, Aberystwyth University, Aberystwyth, SY23 3AR Ceredigion UK; 3 Wales Veterinary Science Centre, Y Buarth, Aberystwyth, SY23 1ND Ceredigion UK; 4 ProTEM Services Ltd, West Sussex, UK; 5 TB Research Group, Animal and Plant Health Agency, New Haw, Addlestone, KT15 3NB Surrey UK; 6 Aberystwyth University, B2.03 Edward Llwyd, Penglais, Aberystwyth, SY23 3FL UK

**Keywords:** Bovine tuberculosis, *Mycobacterium bovis*, Badger, Metabolomics, Diagnostics

## Abstract

**Introduction:**

*Mycobacterium bovis*, the causative agent of bovine tuberculosis (bTB) in cattle, represents a major disease burden to UK cattle farming, with considerable costs associated with its control. The European badger (*Meles meles*) is a known wildlife reservoir for bTB and better knowledge of the epidemiology of bTB through testing wildlife is required for disease control. Current tests available for the diagnosis of bTB in badgers are limited by cost, processing time or sensitivities.

**Materials and Methods:**

We assessed the ability of flow infusion electrospray—high-resolution mass spectrometry (FIE-HRMS) to determine potential differences between infected and non-infected badgers based on thoracic blood samples obtained from badgers found dead in Wales. Thoracic blood samples were autoclaved for handling in a containment level 2 (CL2) hazard laboratory.

**Results:**

Here we show the major differences associated with with *M. bovis* infection were changes to folate, pyrimidine, histidine, glycerophospholipid and phosphonate metabolism.

**Conclusions:**

Our studies have indicated differences in the metabolomic signature of badgers found dead in relation to their infection status, suggesting metabolomics could hold potential for developing novel diagnostics for bTB in badgers. As well as highlighting a potential way to handle samples containing a highly pathogenic agent at CL2 for metabolomics studies.

**Supplementary Information:**

The online version contains supplementary material available at 10.1007/s11306-022-01915-6.

## Introduction

bTB is the most significant infectious disease threat to UK cattle farming (Godfray et al., 2018). bTB was first discovered in the European badger *Meles meles* in Britain in 1971 and since then it has been shown to be endemic and widespread in badgers across the British Isles (Jenkins et al., 2008; Murphy et al., 2010; Schroeder et al., 2020). Badgers, while looking for food during the night, can come into close contact with farmed livestock (Garnett et al., 2002). This behaviour of badgers scavenging for food on farms, combined with the long survival time of *M. bovis* in the environment could facilitate both direct and indirect transmission routes into cattle herds (Ghodbane et al., 2014; Godfray et al., 2018; Krebs et al., 1997; Young et al., 2005). Multiple studies have utilised badgers found dead as a source of disease surveillance information through post-mortem and identification of *M. bovis* (Abernethy et al., 2011; Goodchild et al., 2012; Sandoval Barron et al., 2018; Schroeder et al., 2020)*.*

Eradicating bTB in badger populations requires an accurate test that can monitor infection in this wild animal population. There have been various tests developed over the years for diagnosis of bTB on blood samples from live trapped badgers; PCR-based approaches, the Brock test (indirect ELISA), the BrockTB Stat-Pak assay, IFN-γ immune assays, IgA ELISAs and detection of the P22 multiprotein complex derived from the purified protein derivative (PPD) of bTB by ELISA (Buzdugan et al., 2017; Chambers et al., 2010; Dalley et al., 2019; Infantes-Lorenzo et al., 2019; King et al., 2015). These tests however mostly require time and laboratory equipment to process, which has significant cost and animal welfare implications when dealing with a wild, trapped animal. One solution could be a point-of-care (POC) test, such as that used in Chambers et al. (2010) or Stewart et al. (2017), however with sensitivities quoted as 0.35 and 0.081 respectively, these may have limited use. Therefore, there is the need for a rapid and sensitive POC test for bTB in badgers. The quoted sensitivities of these tests are especially low partly because they are being use to diagnose bTB across the full spectrum of infections. In this study only animals with more marked infections (grossly visible lymph node lesions, which can be cultured) were identified as infected.

Metabolomics is an approach where 1000s of metabolites can be rapidly assessed in a given sample. The challenge of detecting metabolites has been eased in recent years through the development of tools such as high-resolution mass spectrometric platforms and bioinformatic analysis pipelines (Segers et al., 2019). As the metabolome integrates genomic and proteomic changes it may more accurately reflect the status of an organism, organ or cell in response to, for example, pathogenic challenge (Wishart, 2019). As a result, metabolomic approaches offer the possibility of identifying new classes of clinical biomarkers (López-López et al., 2018). Metabolomics has shown promise in the detection of infectious diseases like bTB (Frediani et al., 2014) and is yet to be used in the analysis of samples from badgers with bTB. Metabolomics can be used to initially identify biomarkers that can then be developed into rapid, cheap, POC tests, using a lateral flow system, which can be utilised in the field.

This study aimed to used flow infusion electrospray—high resolution mass spectrometry (FIE-HRMS) to investigate any differences in the thoracic blood of badgers found dead in Wales that are bTB positive and negative based on culture of lymph nodes. As a way of assessing whether this technique could be used to differentiate between samples based on infections status. This is a small number pilot study where samples were all autoclaved prior to extraction. This aimed to facilate studies where researchers only have access to a CL2 lab.

## Materials and methods

### Sample recruitment and collection

The badgers involved in this study were found dead or euthanased at veterinary practices for welfare reasons, as part of an ongoing surveillance study by Welsh Government and the Animal Plant Health Agency (APHA), the All Wales Badger Found Dead study (AWBFD study) (https://gov.wales/badger-found-dead-temporal-and-spatial-mycobacterium-bovis-prevalence-patterns). Badgers reported by members of the public were collected and brought to a laboratory where they were deemed appropriate for post-mortem (PM) if they were intact, not distended with gas, with no severe myiasis and were not frozen. Carcasses spent no more than four days in cold storage before PM. PM involved an external examination; including, weighing, measuring, sexing, approximate aging based on dental wear, and checking for lactation if female. Badgers were scanned for microchips as well as clipping of guard hairs or any colour marker to indicate historical trapping and vaccination. Any signs of external injury, bite wounds, illegal trapping or snaring were also noted. Internal examination was focussed on identification of any gross lesions and samples of tissues were collected for mycobacterial culture. Detailed examination was made of the pericardial sac, lungs, liver and kidneys including internally by making several, longitudinal incisions across each. The following lymph nodes were incised at least once and examined for lesions (submaxillary, retropharyngeal, external cervical, axillary, bronchial, mediastinal, hepatic, gastric, renal (when located), mesenteric, internal iliac, external iliac, superficial inguinal, popliteal). Two pools of samples were then created; pool one contained retropharyngeal, bronchial lymph nodes, mediastinal and hepatic lymph node samples, and pool two contained a section of any bite wound or any internal visible lesions suggestive of bTB. The samples were preserved in 1% aqueous cetylpyridinium chloride and were posted to the APHA laboratory in Starcross, (Devon, UK) for bTB testing. At Starcross the samples were washed in sterile 0.85% saline, then homogenized and inoculated onto six modified Middlebrook 7H11 agar slopes and incubated at 37 °C for up to 12 weeks. Any *M. bovis* that was grown was sent to APHA Weybridge for genotyping. Culture positivity and genotyping provided the basis for designation of badgers as positive.

Two mL of blood was pipetted straight from the badger’s thoracic cavity after removal of the pluck (heart, lungs and trachea). The viscosity, colour and transparency of blood samples varied greatly (Fig. S1); so this blood sample may include other bodily liquids, such as interstitial fluid, lymph and pleural fluid. Samples were frozen at − 80 °C until further analysis. For this study 12 blood samples were chosen to represent six bTB positive and six negative animals with a mix of sex and region (based on the four Welsh TB risk areas; Table S6; Fig. S2). Given the potential that samples may be infected with *M. bovis*, prior to any further processing, samples were thawed and autoclaved at 126 °C for 40 min. The autoclaving process led to a solidification of the samples, producing a solid, rubbery, dark brown substance (the “homogenate”), as well as a small volume (~ 300 μL) of clear, yellow liquid (the “cell lysate”) (Fig. S3).

### Flow infusion electrospray high resolution mass spectrometry (FIE-HRMS)

All samples were processed for metabolomics in a blinded manner; after allocation into groups they were each given a unique identifier number which was unknown to the person processing the samples. Cell lysate and homogenate were analysed separately, for each cell lysate a 200 μL aliquot was used, and for each homogenate sample 0.1 g (± 0.02 g) was used. Samples were added to 1520 μL of 4:1 (v/v) mix of MeOH:chloroform (HPLC grade) and 50 mg of acetone-washed glass beads (< 160 μg, Sigma, UK). Samples were vortexed, shaken (15 min at 4 °C), left to settle (− 80 °C for 20 min) and centrifuged at 1800×*g* for 10 min. 100 μL of the supernatant was transferred to a glass vial with glass insert. Sample injection order was randomised to reduce batch effects and run on an ExactiveTM Orbitrap Mass Spectrometer (Thermo Scientific) as described in Baptista et al. (2018).

### Statistical analysis

Principal Component Analysis (PCA), Partial Least Square Discriminant Analysis (PLS-DA) and Area Under Curve (AUC) analysis were performed using the MetaboAnalyst 4.0 platform (Pang et al., 2021). Raw data were log-transformed and Pareto-scaled before analysis, and outputs were Bonferroni-corrected for multiple comparisons. Major sources of variation were identified using *t-*tests or variable importance projection (VIP) scores. Significant mass:charge ratios (*m/z*) were tentatively identified using the MZedDb database (Draper et al., 2009) based on predicted masses *m/z* identified and likely ionisation forms. In MetaboAnalyst 4.0 pathway analysis (integrating enrichment analysis and pathway topology analysis) incorporating the mummichog algorithm was used for biochemical pathway enrichment analysis (Li et al., 2013). A power analysis reveals that 160 samples per group would be required to give a power of 70%.

## Results

**Fig. 1:**
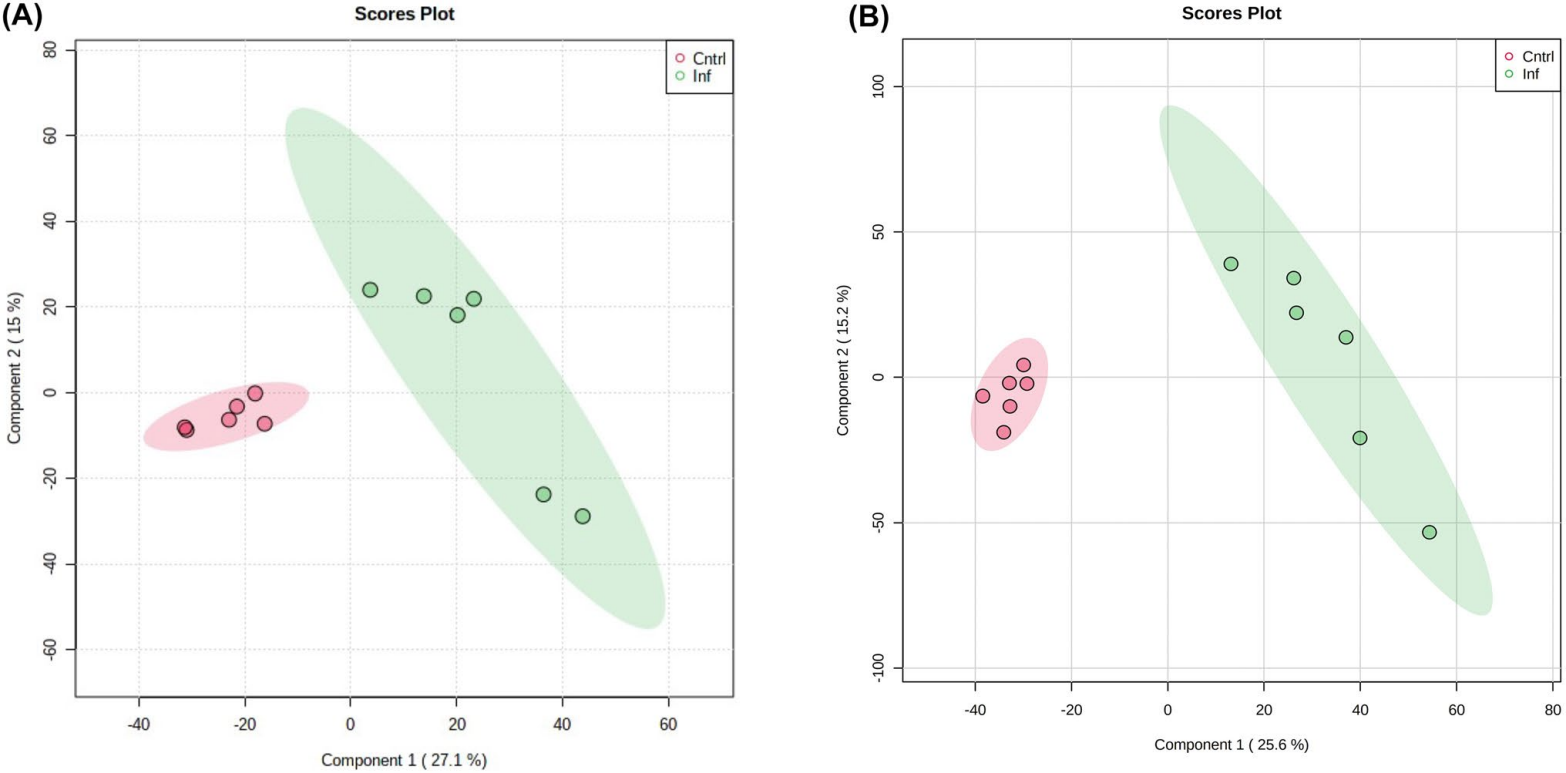
Partial Least Squares Discriminant Analysis of **A** homogenate and **B** cell lysate samples, comparing TB infected (inf) in green and negative badgers (Cntrl) in red. The shaded circles show 95% CI for each group (N = 12)

Samples obtained from the thoracic cavity of PM badgers and autoclaved to be assessable in CL2 laboratories were metabolomically profiled by FIE-HRMS. The derived matrices of identified *m/z* were investigated by multivariate statistics to test if metabolite variation could be linked to *M. bovis* infection status. PLS-DA of the metabolomes of the homogenate or cell lysates following autoclaving showed a clear separation in badgers based on their bTB status (Fig. 1).

**Fig. 2:**
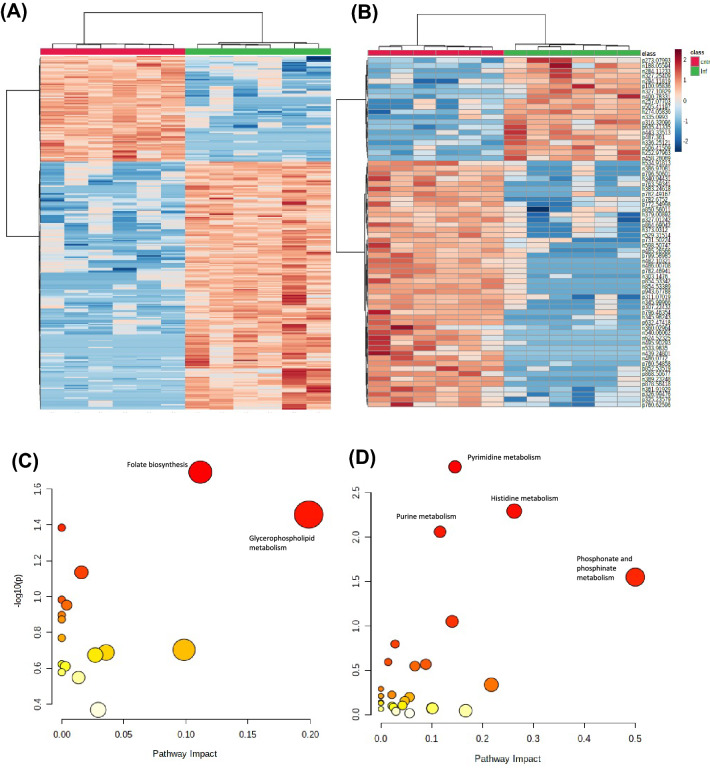
The major sources of variation in homogenates and cell lysates from TB infected and negative badgers. Heatmap of all t-test significant *m/z* values with an AUC of 1 from FIE-MS on the **A** homogenate and **B** cell lysate samples. Hierarchical clustering can be seen with clear separation of the TB positive and TB negative samples (N = 12). The significant *m/z* from **C** homogenates and **D** cell lysates were assessed by pathway enrichment analysis. Y-axis:-log p-values from pathway enrichment analysis. X-axis: pathway impact values from pathway topology analysis. The node colour and radius is based on its p-value and pathway impact values, respectively

The major sources of variation were identified in the homogenate and cell lysates using t-tests and following AUC assessments. Those *m/z* which were significant in t-tests (< 0.05) and had an AUC of 1 are displayed as heat maps (Fig. 2A, B). This univariate display of the *m/z* data indicates consistent discrimination between bTB positive and negative badgers based on increased or decreased *m/z* levels in both the homogenate and cell lysates.

The potential biological relevance of the discriminatory *m/z* were indicated using the mummichog programme which produced both tentative identifications of the *m/z* and linked these to biological pathways (Xia, 2017). These mummichog results were put through further pathway analysis. For the cell lysate, this was based on using t-test significant *m/z* values. However there were too few significant *m/z* values identified by the univariate t-test for the homogenate data so instead variable importance projections (VIPs) from the PLS-DA (Fig. 1) with scores over 1.5 were used. Pathway enrichment assessments on the homogenates indicated significant enrichment of metabolites associated with folate biosynthesis and glycerolphospholipid metabolism (Fig. 2C; Table S1). For the cell lysates, significantly enriched pathways were linked to pyridmidine, purine, histidine and phosphonate and phosphinate metabolism (Fig. 2D; Table S2).

**Fig. 3:**
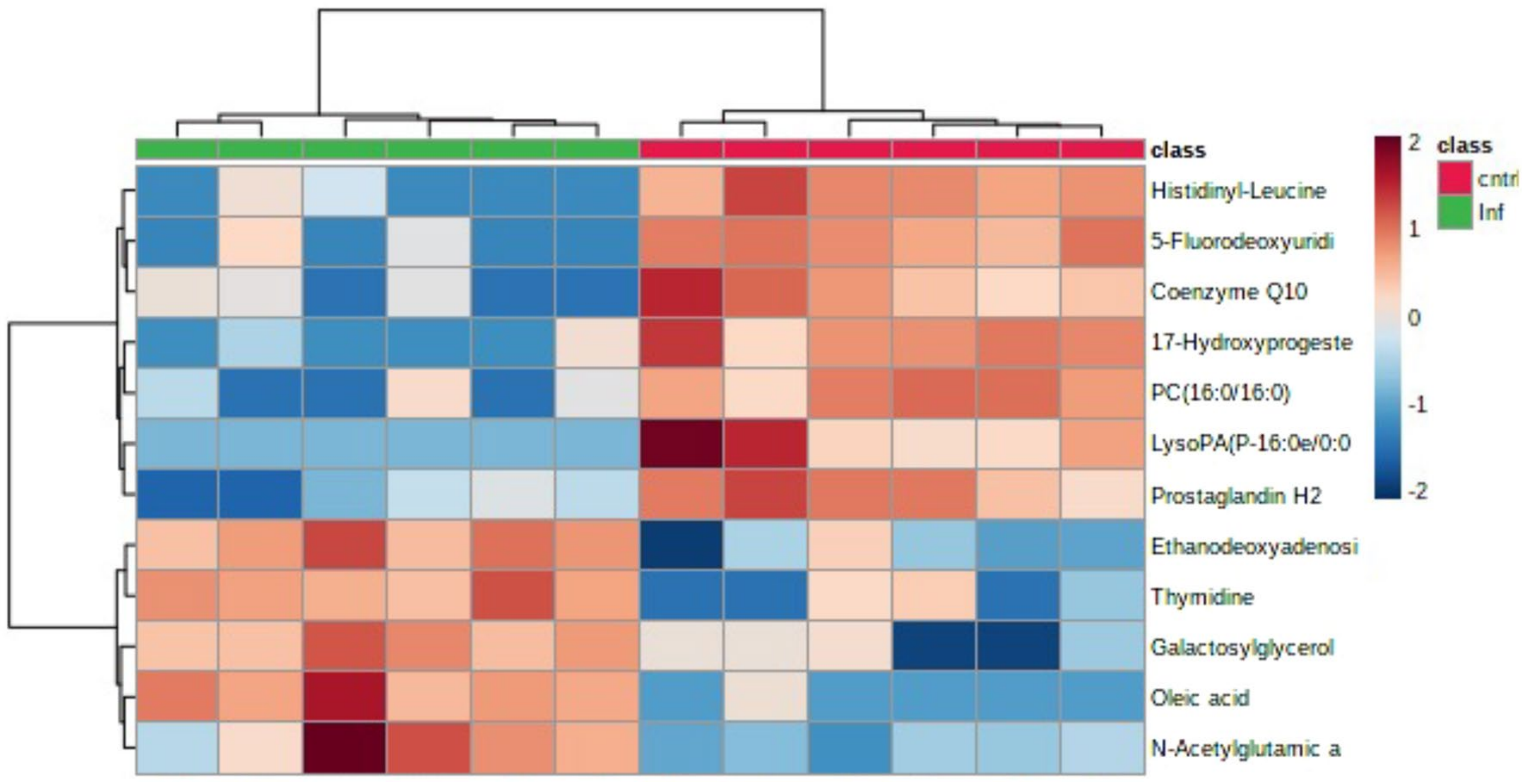
Heatmap of all t-test significant *m/z* values with an AUC of 1 from mummichog algorithm identification of metabolites by FIE-MS on the homogenate samples. ierarchical clustering can be seen with clear separation of the TB positive and TB negative samples (N = 12)

**Fig. 4:**
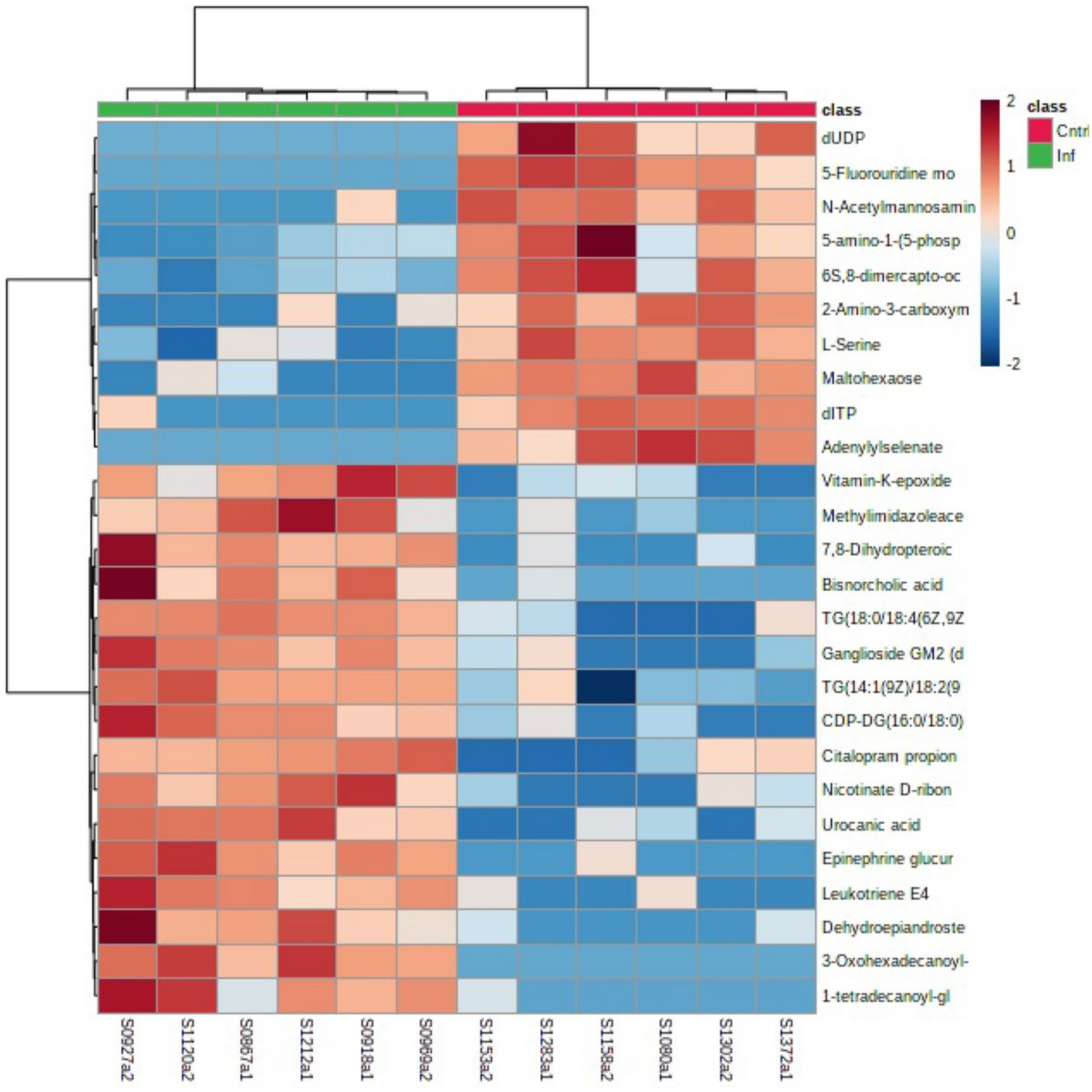
Heatmap of all t-test significant *m/z* values with an AUC of 1 from mummichog algorithm identification of metabolites by FIE-MS on the cell lysate samples. Hierarchical clustering can be seen with clear separation of the TB positive and TB negative samples (N = 12)

The metabolites tentatively identified were also compared using heatmaps. Examining metabolites from the homogenates, bTB positive badgers appeared to show increases in such as the lipid oleic acid, and nucleotide thymidine compared to bTB negative badgers. Equally, decreases were seen in a range of lipids (e.g. PC(18:0/16:0)) and the lipid derivative prostaglandin H2 compared to bTB negative badgers (Fig. 3). The cell lysate data allowed a greater number of metabolites to be tentatively identified (Fig. 4). This is also indicated in a range of lipid changes on *M. bovis* infection, including triacyl glycerols (TG), ganglioside GM2 and leukotriene E4. Changes were also noted as linked to nucleotide metabolism for example, dITP and dUDP.

## Discussion

bTb in UK cattle remains a significant disease burden and its presence in wild badgers and ability to spread from them to cattle, and vice versa, is now widely accepted (Godfray et al., 2018). Having access to better testing for badgers, specifically accurate and fast POC testing, could help significantly improve understanding of the epidemiology of this disease process and aid the control of it. Our pilot study assessed whether thoracic blood could indicate the bTB status of the badger, and thus represents the first step in a programme which could lead to a POC test for bTB badgers based on peripheral blood sampling of live animals.

Our data indicated differences in the metabolomes of thoracic blood samples collected from badgers found dead when infected with *M. bovis*. These samples were autoclaved to allow the manipulation of samples safely at CL2. Although this could have introduced an element of artifactuality to our results, the samples were treated the same whether from bTB positive or negative badgers. Therefore, it is likely that the observed differences reflected, directly or indirectly, metabolomic differences linked to *M. bovis* infection. This would benefit from being further assessed within a CL3 setting by comparing non-autoclaved bTB positive and negative blood samples, which we do not have access to. Biological aspects such as age, sex and bTB risk area (Table S3) were matched, where possible from the small number of available bTB positive samples, across the two groups. So any changes seen between groups area more likely associated with their disease status (*M. bovis* culture positive or negative).

The sample size is small in this study (N = 12), so that there is risk of confounding factors and low statistical power. To mitigate against this we focused on variables with AUC values of 1, leading to a total of 295 targeted *m/z* in cell lysate and 68 m*/z* in homogenate samples. If further work was carried out, a larger sample size would be required to highlight fewer, more statistically robust *m/z* peaks. According to our power analysis, 160 samples per group would give a power of 70%, highlighting the greatly under-powered nature of this pilot study. So due to access to only low numbers of samples here we aim to mainly draw conclusions about pathways affected more generally. Each individual *m/z* value detected by MS has multiple potential identifications, depending on the size and ionisation of the metabolite. The mummichog algorithm looks at all the *m/z* values identified in a dataset and attempts to link *m/z*’s that appear many times in specific pathways. From these some inferences can then be drawn about biological processes occuring in the host. For instance the pathway analysis shows significant fatty acid changes being identified with *M. bovis* infection. These can be linked to the processing of inflammatory mediators, such as prostaglandins, which were also targeted in our analyses. There are also changes associated with differential sugar processing. Some pathways identified such as histidine, pyrimidine and purine metabolism have previously been identified as novel biomarkers for tuberculous disease in humans and animal models, and are also shown to change with active tuberculosis (TB) or in patients following treatment (Shin et al., 2011; Tientcheu et al., 2015; Vrieling et al., 2019; Weiner et al., 2012; Yi et al., 2019). Folate biosynthesis is known to be important in TB metabolism in humans, and has been a source of interest for anti-microbial treatment more generally (Bermingham & Derrick, 2002). Glycerophospholipid metabolism has also been identified as altering significantly in tuberculosis (Zhong et al., 2016). However, given the low sample size and the resulting lack of statistical power, these observations require validation in further analyses.

## Conclusion

Comparison of thoracic blood samples collected from badgers found dead in Wales reveals differences in the metabolomes of *M. bovis* infected badgers. Mass spectrometry provides potential new insights into diagnosing bTB in this species. If differences can still be accurately identified following aggressive heat treatment such as autoclaving then this opens up the potential for samples containing this organism to be investigated in a CL2 setting. Further work is required with a much larger sample size to more accurately identify pathways and metabolites that are associated with *M. bovis* infection. Should the same changes be consistently identified across a larger sample size study, and also be confirmed in a CL3 based experiment with un-autoclaved blood, and then validated in blood samples from living badgers, such metabolomic signatures could provide a novel way of diagnosing bTB on blood samples from trapped badgers.

## Supplementary Information

Below is the link to the electronic supplementary material.

Supplementary file1 (PDF 1635 kb)

## Data Availability

The raw data are available on Mendeley Data (https://doi.org/10.17632/4tt9ps4zhj.1). All sample metadata used in this study is provided in Supplementary Data.

## Abbreviations

bTB: Bovine tuberculosis
FIE-HRMS: Flow infusion electrospray-high-resolution mass spectrometry
PLSDA: Partial least squares discriminant analysis
PCA: Principal component analysis
VIP: Variable importance projection

## Declarations

### Welfare of animals

All samples used in this study were collected from badgers that were found dead. The carcasses were undergoing a post-mortem as part of an ongoing Welsh government study (All Wales Badger Found Dead study) to monitor levels of tuberculosis in the population, when an additional sample was taken for this study.
