# Supplementary material for: Untargeted metabolomic analysis of thoracic blood from badgers indicate changes linked to infection with bovine tuberculosis (Mycobacterium bovis): a pilot study
Source: Metabolomics. 2022 Jul 27;18(8):61. doi: 10.1007/s11306-022-01915-6 (PMC9329164; doi:10.1007/s11306-022-01915-6)
Supplement: Supplementary file 1 — Supplementary file1 (PDF 1635 kb) [file 11306_2022_1915_MOESM1_ESM.pdf]

Untargeted metabolomic analysis of thoracic blood from badgers indicate changes linked to infection with bovine tuberculosis (*Mycobacterium bovis*)

James Scott Bauman[1], Richard Pizzey[1], Manfred Beckmann[1], Bernardo Villarreal-Ramos[1, 2, 5]
Jonathan King[3], Beverley Hopkins[3], David Rooke[4], Glyn Hewinson[1,2], Luis A. J. Mur[1, *]

[1] Aberystwyth University, Institute of Biological, Environmental and Rural Science, Aberystwyth, Ceredigion, SY23 3DA, UK

[2] Centre of Excellence for Bovine Tuberculosis, Aberystwyth University, Ceredigion, SY23 3AR, UK

[3] Wales Veterinary Science Centre, Y Buarth, Aberystwyth, Ceredigion, SY23 1ND, UK

[4] ProTEM Services Ltd, West Sussex, UK

[5]Animal and Plant Health Agency, TB Research Group, New Haw, Addlestone, Surrey, KT15 3NB.

Corresponding author:
Prof. Luis Mur
B2.03 Edward Llwyd
Penglais
Aberystwyth
SY23 3FL
tel:+44 1970 622981

Supplementary material

28

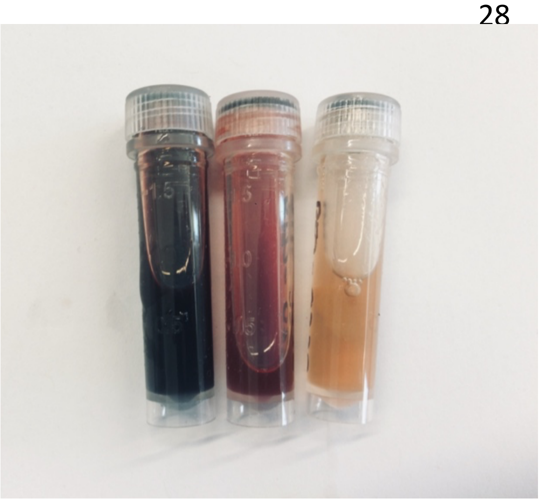

Figure S1: The highly variable nature of the thoracic 'blood samples' collected, prior to autoclaving

Table S1: Pathway identification based on mummichog algorithm alone, using VIPs >1.5 from homogenate PLSDA data

Pathway identified	Match status	P value
**Galactose metabolism**	14/27	5.43×10^{-06}
**Pentose phosphate pathway**	11/22	9.12×10^{-05}
**Amino sugar and nucleotide sugar metabolism**	14/37	4.1×10^{-04}
**Starch and sucrose metabolism**	9/18	4.15×10^{-04}
**Glycolysis / Gluconeogenesis**	11/26	5.75×10^{-04}
**Butanoate metabolism**	6/15	0.015175
**Citrate cycle (TCA cycle)**	7/20	0.019568
**Arginine biosynthesis**	5/14	0.043377
**Alanine, aspartate and glutamate metabolism**	8/28	0.043442
**D-Glutamine and D-glutamate metabolism**	3/6	0.045157

Table S2: Pathway identification based on mummichog algorithm, using t-test significant ($p<0.05$) *m/z* values from cell lysate data

Pathway identified	Match status	P value
**Pyrimidine metabolism**	7/39	0.001622
**Histidine metabolism**	4/16	0.005098
**Purine metabolism**	8/65	0.008724
**Phosphonate and phosphinate metabolism**	2/6	0.028275
**Amino sugar and nucleotide sugar metabolism**	4/37	0.088918

Table S3: Badger sample metadata

Badger ID	Sex	Age	*M. bovis* culture	Welsh Risk Area
**1080**	M	adult	Negative	WLRA
**1153**	M	adult	Negative	WHRE
**1158**	F	adult	Negative	WHRE
**1283**	M	adult	Negative	WIA2
**1208**	M	adult	Negative	W_IA2
**1372**	M	adult	Negative	WIA2
**867**	F	adult	Positive	WIA2
**869**	M	adult	Positive	WHRE
**918**	M	adult	Positive	WHRE
**927**	M	adult	Positive	WHRE
**1120**	F	adult	Positive	WHRE
**1212**	M	adult	Positive	WIA2

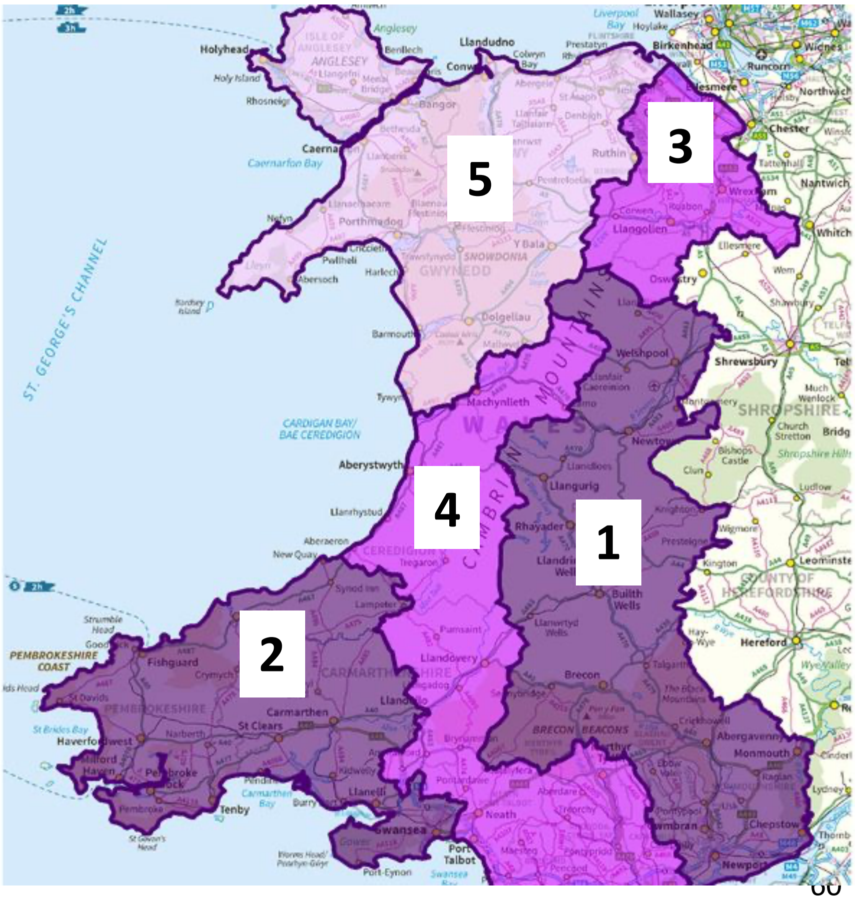

Figure S2: **Welsh bTB Risk Areas**

1)High risk east (HRE), 2) High risk west (HRW), 3) Intermediate TB North (IA1), 4) Intermediate TB Mid (IA2), 5) Low risk area (LRA)

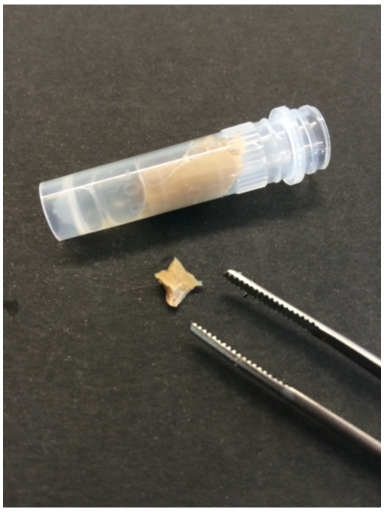

Figure S3:

The solidified 'homogenate' produced by autoclaving the thoracic blood samples
